# Functional and structural impact of the most prevalent missense mutations in classic galactosemia

**DOI:** 10.1002/mgg3.94

**Published:** 2014-06-23

**Authors:** Ana I Coelho, Matilde Trabuco, Ruben Ramos, Maria João Silva, Isabel Tavares de Almeida, Paula Leandro, Isabel Rivera, João B Vicente

**Affiliations:** 1 Metabolism and Genetics Group, Research Institute for Medicines and Pharmaceutical Sciences (iMed.UL), Faculty of Pharmacy, University of Lisbon1649-003, Lisbon, Portugal; 2 Department of Biochemistry and Human Biology, Faculty of Pharmacy, University of Lisbon1649-003, Lisbon, Portugal

**Keywords:** Chemical/pharmacological chaperones, classic galactosemia, GALT, misfolding, missense mutations, protein aggregation, proteostasis regulators.

## Abstract

Galactose-1-phosphate uridylyltransferase (GALT) is a key enzyme in galactose metabolism, particularly important in the neonatal period due to ingestion of galactose-containing milk. GALT deficiency results in the genetic disorder classic galactosemia, whose pathophysiology is still not fully elucidated. Whereas classic galactosemia has been hypothesized to result from GALT misfolding, a thorough functional–structural characterization of GALT most prevalent variants was still lacking, hampering the development of alternative therapeutic approaches. The aim of this study was to investigate the structural–functional effects of nine *GALT* mutations, four of which account for the vast majority of the mutations identified in galactosemic patients. Several methodologies were employed to evaluate the mutations' impact on GALT function, on the protein secondary and tertiary structures, and on the aggregation propensity. The major structural effect concerns disturbed propensity for aggregation, particularly striking for the p.Q188R variant, resulting from the most frequent (∼60%) allele at a worldwide scale. The absence of major effects at the secondary and tertiary structure levels suggests that the disturbed aggregation results from subtle perturbations causing a higher and/or longer exposure of hydrophobic residues in the variants as compared to WT GALT. The results herein described indicate a possible benefit from introducing proteostasis regulators and/or chemical/pharmacological chaperones to prevent the accumulation of protein aggregates, in new avenues of therapeutic research for classic galactosemia.

## Introduction

Classic galactosemia (OMIM #230400) is an autosomal recessive disorder caused by mutations in the *GALT* gene, resulting in deficient activity of galactose-1-phosphate uridylyltransferase (GALT, EC 2.7.7.12), a key enzyme in galactose metabolism (Fridovich-Keil and Walter 2008). GALT catalyzes the second step of the Leloir pathway, converting galactose-1-phosphate (Gal-1-P) and uridine diphosphate (UDP)-glucose (UDP-Glc) into glucose-1-phosphate and UDP-galactose (UDP-Gal) (Fridovich-Keil and Walter 2008).

In classic galactosemia, acute symptoms generally appear soon after birth upon exposure to milk, and include the following: vomiting, diarrhea, excessive weight loss, lethargy, hypotonia, liver dysfunction, and, in the absence of intervention, can escalate to cataracts, *Escherichia* (*E*.) *coli* sepsis, and eventually to neonatal death (Holton et al. 2001; Bosch 2006; Suchy et al. 2007; Fridovich-Keil and Walter 2008). These symptoms generally self-resolve once the patient is placed on a stringent lifelong dietary restriction of galactose, which is the current standard of care (Fridovich-Keil 2006). However, despite resolving the acute and potentially lethal symptoms, the dietetic treatment does not prevent the development of serious long-term complications, namely cognitive and neurologic disabilities, and premature ovarian insufficiency in females (Waggoner et al. 1990; Fridovich-Keil and Walter 2008).

Thus far, 266 variations have been described at the *GALT locus* (available at http://www.arup.utah.edu/database/GALT/GALT_display.php, last surveyed on December 2013), of which missense mutations constitute the majority (>60%), despite the high allelic heterogeneity (Calderon et al. 2007). In particular, the c.563A>G transition, originating the p.Q188R variant, is by far the most frequent, accounting for ∼63% of *GALT* mutant alleles (Elsas et al. 1995; Tyfield et al. 1999). Its incidence is particularly high in European descendant patients, reaching >90% of mutant alleles in Ireland; it has, however, never been reported in Asian descendant patients (Hirokawa et al. 1999; Fridovich-Keil and Walter 2008; Coss et al. 2013). Other frequent mutations originate the p.S135L, p.K285N, and, p.N314D variants. The c.404 C>T mutation (p.S135L) affects mostly African descendant patients, ranging from approximately half of mutant alleles in African Americans to ∼90% in South African patients (Elsas et al. 1995; Tyfield et al. 1999). The second most frequent *GALT* mutant allele in European descendant patients is c.855G>T (p.K285N), with a higher incidence in Eastern Europe, reaching 34% in Poland (Elsas et al. 1995; Tyfield et al. 1999; Zekanowski et al. 1999; Suzuki et al. 2001). The c.940A>G mutation (p.N314D) appears to be an evolutionary remnant, as the D314 is actually the ancestral variant that persists nowadays at a pan-ethnic frequency of nearly 10% (Suzuki et al. 2001; Carney et al. 2009). This missense variation is associated with two variant forms depending on the presence of additional base changes: the Los Angeles variant, which carries p.N314D in *linkage disequilibrium* with p.L218L (c.652C>T; CTA→TTA) (Langley et al. 1997); and the Duarte variant, which carries p.N314D in *linkage disequilibrium* with three intronic variations (c.378-27G>C, c.507+62G>A, c.508-24G>A), and a deletion in the *GALT* promoter (c.-119_-116delGTCA) (Elsas et al. 2001; Trbusek et al. 2001; Carney et al. 2009). Functional studies revealed that the translation favorable codon TTA from p.L218L (Langley et al. 1997) and the promoter deletion (Trbusek et al. 2001) are respectively responsible for the altered activities of the Los Angeles and Duarte variants, and that the p.N314D variation is actually not a disease-causing mutation and is better considered a polymorphism.

**Figure 1:**
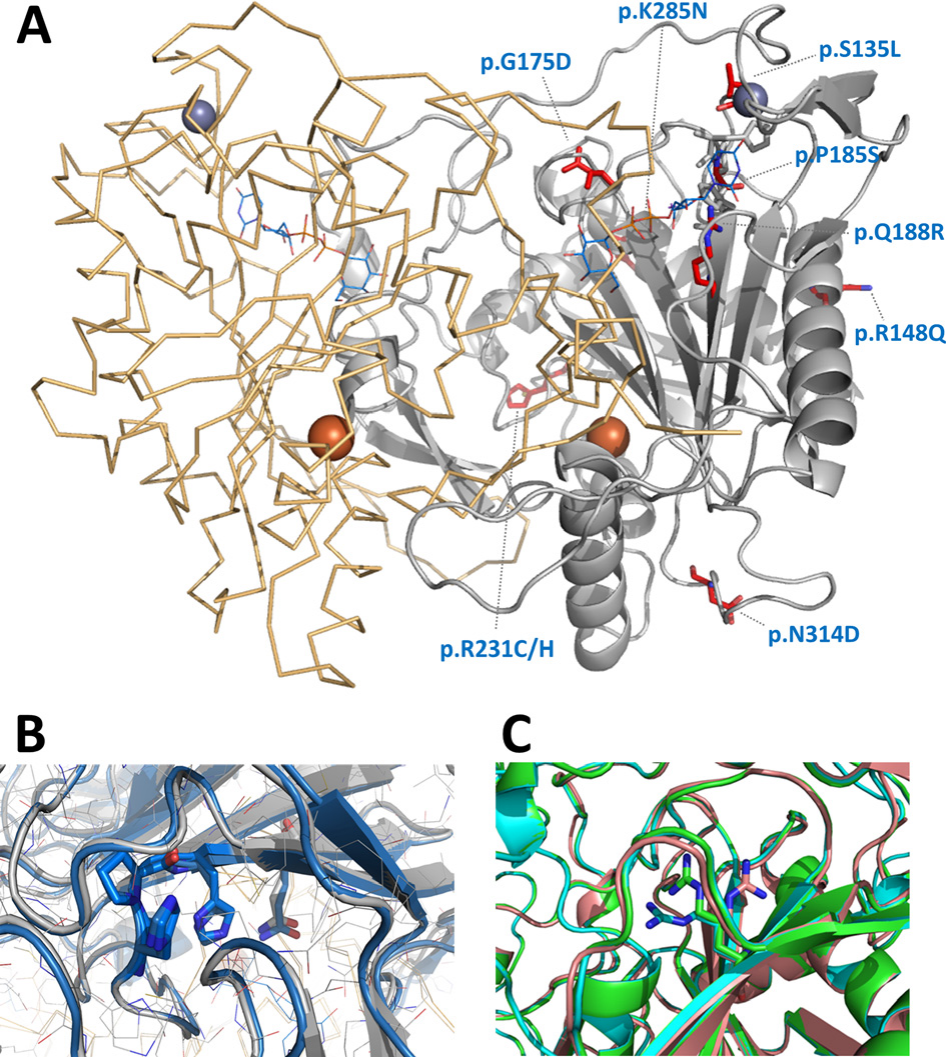
Structural models of GALT dimer. (A) Structural model of human GALT (grey cartoon representation) obtained using *Escherichia coli* GalT crystallographic structure as template (orange ribbon representation, PDB ID: 1GUP). Iron (orange sphere), zinc (purple sphere), and bound UDP-glucose (light blue lines) originate from 1GUP PDB. Variants herein studied are represented in red sticks. Panel B, structural models of WT human GALT, generated with 1GUP (grey) or 1R3A (blue) as templates, aligned with UCSF Chimera (Pettersen et al. 2004) (RMSD 0.403 Å). Sticks show the overlap between the side-chains from the active site residues H184-P185-H186 and the residue Q188, validating the generated models. (C) Structural models of human GALT variant p.Q188R, generated with 1GUP (pink) or 1R3A (blue) as templates, and downloaded from the GALT Protein Database 2.0 server (http://bioinformatica.isa.cnr.it/GALT/index0.html) (green); models aligned with UCSF Chimera (Pettersen et al. 2004) (RMSD's between 0.088 Å and 0.403 Å); zoom-in into the location of the amino acid substitution (sticks show the predicted position of the R188 side-chain). Figure generated with PyMOL. GALT, galactose-1-phosphate uridylyltransferase.

Notwithstanding the several studies on the molecular basis of these mutations' pathogenicity (Reichardt et al. 1991; Fridovich-Keil and Jinks-Robertson 1993; Fridovich-Keil et al. 1995a,b; Lai et al. 1996, 1998, 1999; Langley et al. 1997; Wells and Fridovich-Keil 1997; Lai and Elsas 2001; Riehman et al. 2001; Chhay et al. 2008), a characterization of these variants focusing on different structural features is still missing. To date, there is no solved three-dimensional structure of the human GALT. However, based on the availability of the *E. coli* GalT structure (Wedekind et al. 1995) and on the high sequence identity and similarity between the human and the prokaryotic GALT, a three-dimensional model of the human GALT was herein generated (Fig.1A and B), as previously reported (Marabotti and Facchiano 2005), which provided important insights into the structural and functional features of this protein. GALT is a member of the transferase branch of the histidine triad (HIT) family of enzymes; the catalytic site sequence His-Pro-His is conserved in nature, and was firstly identified in the *E. coli* enzyme at residues 164–166, corresponding to residues 184–186 in the human sequence (Wedekind et al. 1995; Brenner 2002; Leslie 2003; Marabotti and Facchiano 2005). The reaction displays ping-pong kinetics and a double displacement mechanism, involving an uridylyl-enzyme, in which the nucleophilic histidine at residue 186 is transiently nucleotidylated (Wong and Frey 1974; Field et al. 1989; Wedekind et al. 1995). The active enzyme is an 86.6-kDa homodimer (Fig.1A), with two active sites, each formed by residues contributed by both subunits (Thoden et al. 1997). Whereas the *E. coli* GalT has two mononuclear metal-binding sites (one for zinc and the other for iron) with proposed structural roles, the human GALT lacks two of the zinc ligands, thus it remains to be established whether metal binding in the human protein is comparable to that of the bacterial GalT (Geeganage and Frey 1999).

A recent study reported that five missense mutations in the *GALT* gene led to misfolding of the resulting GALT variants, suggesting classic galactosemia as a conformational disorder (McCorvie et al. 2013). However, little is known about the conformational impact of the most prevalent mutations, which hampers the design of alternative therapies for this monogenic disorder based on the use of stabilizing low-molecular-weight compounds (Leandro and Gomes 2008). Accordingly, the aim of this study was to further investigate the structural–functional effects of the most prevalent mutations in the *GALT* gene, originating the p.Q188R, p.S135L, p.K285N, and p.N314D variants, and of five other clinically relevant mutations (variants p.R148Q, p.G175D, p.P185S, p.R231C, and p.R231H).

## Materials and Methods

### Production of recombinant human GALT variants

Recombinant human GALT was produced by cloning the human *GALT* cDNA (GenBank ID M60091.1; a kind gift of Prof. Judith Fridovich-Keil, Emory University, Atlanta, GA) into the HindIII and SalI restriction sites of pET24b(+), the T7 tag being deleted using the NdeI and EcoRI enzymes. Four CAT and two CAC codons were then inserted to introduce an N-terminal hexa-histidyl tag, by site-directed mutagenesis (QuikChange® II XL Mutagenesis kit; Stratagene, La Jolla, CA), using the primers 6His-F (CCAGCGGATCCCCCTCAAAAATGCATCATCACCATCACCACATGTCGCGCAGTGGAACCGATC) and 6His-R (GATCGGTTCCAC TGCGCGACATGTGGTGATGGTGATGATGCATTTTTGAGGGGGATCCGCTGG). All changes were confirmed by sequencing in both orientations. Site-directed mutagenesis with the NZY mutagenesis kit (NZYTech, Lisbon, Portugal) was employed to introduce all the mutations herein under study using the primers listed in Table S1. Direct sequencing in both forward and reverse orientations was used to confirm the correct introduction of mutations and to exclude additional mutational events.

Vectors bearing the cDNA encoding the GALT variants were transformed into *E. coli* BL21 (DE3) Rosetta cells. For protein production, cells were grown in M9 minimal medium (Maniatis et al. 1982) supplemented with 100 *μ*mol/L ferrous ammonium sulfate and 100 *μ*mol/L zinc sulfate, at 37°C. Protein expression was induced by addition of 400 *μ*mol/L isopropyl β-D-1-thiogalactopyranoside once the Abs_600nm_ reached 0.3, the cultures were placed at 21°C, and the cells were harvested after 4 hours. Bacterial cells were resuspended in buffer A (50 mmol/L Tris-HCl pH 7.5, 300 mmol/L KCl, and 10% glycerol; used throughout for protein handling) with 1 mg/mL lysozyme and 500 *μ*mol/L phenymethanesulfonyl fluoride, disrupted by sonication, and clarified by centrifugation (5 min at 8000*g*).

The fusion proteins were purified by immobilized metal affinity chromatography (IMAC), by loading the cellular extracts into a 1-mL FF-Crude column (Amersham, GE Healthcare, Uppsala, Sweden) and eluting the proteins with buffer A containing increasing concentrations of imidazole (pure GALT eluted at 500 mmol/L imidazole). After purification, imidazole was eliminated with a desalting column pre-equilibrated and eluted with buffer A, and protein solutions were concentrated by ultrafiltration, aliquoted, flash-frozen in liquid nitrogen and stored at −80°C. Protein purity was assessed by SDS-PAGE, and protein concentration was determined by the Bradford assay using bovine serum albumin as the protein standard (Bradford 1976).

### GALT activity assays and thermal inactivation profiles

GALT enzymatic activity was measured as previously described (Lindhout et al. 2010), and performed on the same day as purification. All assays were carried out for 30 min at 37°C, in a reaction mixture containing 2.0 mmol/L Gal-1-P, 0.5 mmol/L UDP-Glc, 40 *μ*mol/L dithiothreitol (DTT) and 125 mmol/L glycine, in 40 mmol/L Tris-HCl, pH 7.5. UDP-Glc and UDP-Gal were separated by high performance liquid chromatography (HPLC) and analyzed by UV detection at 262 nm (Lindhout et al. 2010; Coelho et al. 2013). The enzyme activity was expressed in *μ*mol UDP-Gal formed per hour per mg protein at 37°C (*μ*mol UDP-Gal h^−1^ mg^−1^). Adequate controls lacking either substrate or the GALT protein were routinely performed. Wild-type (WT) GALT kinetic parameters for UDP-Glc and Gal-1P were determined in the same conditions as in (Coelho et al. 2013) with minor modifications namely the use of nine concentrations of UDP-Glc (0.02–1.5 mmol/L; [Gal-1-P] = 2.0 mmol/L), and nine concentrations of Gal-1-P (0.05–6.0 mmol/L; [UDP-Glc] = 0.5 mmol/L). The steady-state kinetic constants were determined by nonlinear regression analysis using the GraphPad Prism 6 software (GraphPad, Software, Inc., La Jolla, CA), the Michaelis–Menten equation for Gal-1-P and the modified Michaelis–Menten equation to account for substrate inhibition for UDP-Glc.

Thermal inactivation profiles were obtained by analyzing enzyme activity as a function of temperature in the 20–65°C range. Aliquots of protein (WT and p.N314D: 4.28 *μ*g/mL; remaining variants: 21.4 *μ*g/mL) were incubated at the different temperatures for 10 min, immediately chilled on ice for 10 min, and enzyme activity was determined by adding the reaction mixture described above and incubating at 37°C for 30 min. Enzymatic activity values plotted as a function of temperature yielded sigmoidal curves, from which the midpoints of thermal inactivation (*T*_½_) were obtained from the inflexion point. Two assays were performed for each temperature data point, and the WT GALT thermal inactivation profile was repeated in parallel with each tested variant.

### Far-UV circular dichroism spectropolarimetry

Far-UV circular dichroism (far-UV CD) spectra and thermal denaturation profiles were recorded in a Jasco J-710 spectropolarimeter (Easton, MD), coupled to a Jasco PTC-348WI Peltier temperature controller and a Haake G/D8 water bath (Thermo-Fisher Scientific, Waltham, MA). All GALT protein samples were at 0.15 mg/mL, each spectrum being the result of six accumulations at a 50 nm/min scan rate, at 20°C, in a 0.1 cm light path cuvette. Thermal denaturation profiles were obtained by monitoring molar ellipticity at 222 nm, in the 20–90°C temperature range (1°C/min slope; data pitch: 1°C; delay time: 0 sec). Temperature scan curves were fitted to a two-state model.

### Differential scanning fluorimetry

Differential scanning fluorimetry (DSF) is a methodology whereby a fluorescent dye binds to the proteins buried hydrophobic residues that become exposed upon thermal unfolding. DSF assays were performed in a C1000 Touch thermal cycler equipped with a CFX96 optical reaction module (Bio-Rad, Hercules, CA), by having the GALT variants at a 0.1 mg/mL (∼2.5 *μ*mol/L in monomer) final concentration in buffer A, SYPRO orange (Invitrogen Corporation, Carlsbad, CA) at a 5× working concentration (Niesen et al. 2007), in a 50 *μ*L total volume. A 10-min incubation step at 20°C preceded the temperature ramp from 20 to 90°C at 1°C/min, with a 1-sec hold time every 0.2°C and fluorescence acquisition using the HEX channel (excitation maximum at 535 nm, emission maximum at 555 nm). Assays using 2.0 mmol/L Gal-1-P, 0.5 mmol/L UDP-Glc, 100 *μ*mol/L Fe^2+^, and 100 *μ*mol/L Zn^2+^ were also performed. Control assays in the absence of protein were routinely performed. Data were processed using CFX Manager software V3.0 (Bio-Rad). Temperature scan curves were fitted to a biphasic sigmoidal function and the *T*_m_ values were obtained from the inflexion points of the first and second transitions. Variations in *T*_m_ values are considered significant when |Δ*T*_m_| ≥ 2°C (above the standard deviation).

### Dynamic light scattering

Dynamic light scattering (DLS) data were acquired in a ZetaSizer Nano-S (Malvern Instrument, Malvern, UK) particle size analyzer, coupled to a Peltier temperature control unit, using a He–Ne laser as the light source (633 nm). Prior to data collection, protein samples were centrifuged at 15,000*g* for 30 min at 4°C, diluted in buffer A to a final concentration of 0.15 mg/mL, and filtered with a 0.22 *μ*m membrane to remove large aggregates. Temperature was ramped from 20°C to 70°C at 0.5°C/min, with the particle size average, distribution, and total scattering intensity being collected. Data were processed using Zetasizer Nano DTS software v7.01 (Malvern Instrument). The aggregation temperature (*T*_agg_), defined as the temperature at which both size and intensity start to increase significantly, was determined by fitting the obtained data to a plateau followed by one phase association equation. The kinetics of thermal aggregation was monitored at 37°C and 42°C for 60 min. By plotting light scattering intensity as a function of time, sigmoidal curves were obtained and the *t*_1/2_ was determined as the time elapsed to reach half saturation of aggregated protein in the sample.

### In silico analysis

Structural models of human GALT, based either on the *E. coli* GalT structure (PDB ID: 1GUP) or on the structural model of human GALT reported in (Marabotti and Facchiano 2005) (PDB ID: 1R3A), were obtained from the Swiss-Model server (Arnold et al. 2006; Kiefer et al. 2009). To obtain the structural models of the variants with the same methodology, the mutated sequences were submitted. The obtained structural models were aligned with UCSF Chimera (Pettersen et al. 2004), using the Needleman-Wunsch algorithm with default settings. Comparative analysis of the structural models and the corresponding electrostatic surface maps was done with the PyMOL software (DeLano Scientific, San Carlos, CA).

## Results

### Impaired catalytic ability of GALT mutants

The WT recombinant human GALT was isolated in its active state, displaying a *V*_max_ of 59.1 *μ*mol UDP-Gal h^−1^ mg^−1^ and a *K*_M_ of 1.08 mmol/L for Gal-1-P, and a *V*_max_ of 75.5 *μ*mol UDP-Gal h^−1^ mg^−1^ and a *K*_M_ of 425 *μ*mol/L for UDP-Glc.

Aside from the p.N314D variant, which displayed nearly identical enzymatic activity to the WT protein, all the studied GALT variants presented markedly reduced (≤0.2% of WT for p.Q188R, p.S135L, and p.G175D) or apparently null enzymatic activity, that is, below the assay detection limit (Table1). Thermal inactivation profiles were obtained for the GALT variants exhibiting measurable catalytic activity. All the analyzed variants, namely p.Q188R, p.S135L, p.N314D, and p.G175D, displayed lower *T*_½_ than that of the WT GALT, with Δ*T*_½_ ranging from −8.1°C to −19.9°C (Table1).

**Table 1:** Structural and functional parameters determined for recombinant WT and mutant GALT

	Enzyme activity	Thermal inactivation	Circular dichroism	Differential scanning fluorimetry		Dynamic light scattering		
				Thermal denaturation		Thermal aggregation	Aggregation kinetics	
							37°C	42°C
	(% WT)	*T*_½_ (°C)	*T*_m_ (°C)	*T*_m1_ (°C)	*T*_m2_ (°C)	*T*_agg_ (°C)	*t*_½_ (min)	*t*_½_ (min)
WT	100	55.5 ± 3.2	53.0 ± 1.5	43.7 ± 0.7	52.4 ± 1.2	41.3 ± 0.1	26.6 ± 0.1	7.0 ± 0.3
Q188R	0.2^1^	46.6 ± 3.3	56.4 ± 0.9	42.0 ± 0.4	52.3 ± 0.3	37.4 ± 0.2	6.7 ± 0.1	2.3 ± 0.1
S135L	0.1^2^	37.3 ± 2.1	54.4 ± 1.0	44.2 ± 0.1	51.8 ± 0.2	41.3 ± 0.1	36.0 ± 1.4	6.9 ± 0.1
K285N	n.d.^3^	n.a.	55.9 ± 0.3	42.7 ± 0.7	51.4 ± 0.2	41.2 ± 0.1	27.9 ± 1.0	7.5 ± 0.1
N314D	101	47.4 ± 2.2	56.1 ± 0.7	43.8 ± 0.1	53.9 ± 0.1	41.4 ± 0.1	41.8 ± 6.3	6.7 ± 0.1
R148Q	n.d.^3^	n.a.	55.0 ± 0.9	44.3 ± 0.2	53.3 ± 0.4	40.1 ± 1.0	28.4 ± 8.1	5.0 ± 0.4
G175D	0.2^4^	35.6 ± 3.1	54.4 ± 0.3	43.1 ± 0.1	51.3 ± 0.1	40.4 ± 0.5	15.0 ± 0.1	3.6 ± 0.1
P185S	n.d.^3^	n.a.	55.6 ± 0.1	42.9 ± 0.5	52.1 ± 0.4	41.0 ± 0.1	10.4 ± 0.1	3.3 ± 0.4
R231C	n.d.^3^	n.a.	56.7 ± 3.5	43.9 ± 0.2	52.1 ± 0.1	42.0 ± 0.1	48.3 ± 9.8	7.6 ± 0.1
R231H	n.d.^3^	n.a.	52.6 ± 3.8	42.4 ± 0.1	51.0 ± 0.4	41.0 ± 0.1	24.7 ± 0.6	4.9 ± 0.1

Enzyme activity and thermal inactivation profiles determined by HPLC; secondary structure probed by far-UV circular dichroism; tertiary structure analyzed by differential scanning fluorimetry; aggregation propensity studied by dynamic light scattering.

1 Highest detected activity: 141 nmol UDP-Gal h^−1^ mg^−1^.

2 Highest detected activity: 70 nmol UDP-Gal h^−1^ mg^−1^.

3 Below the detection limit of the assay (6.1 nmol UDP-Gal h^−1^ mg^−1^; reaction carried out with 4.8 or 21.4 *μ*g/mL of protein for 1 h).

4 Highest detected activity: 112 nmol UDP-Gal h^−1^ mg^−1^.

### Limited impact of GALT mutations on the secondary and tertiary structure

**Figure 2:**
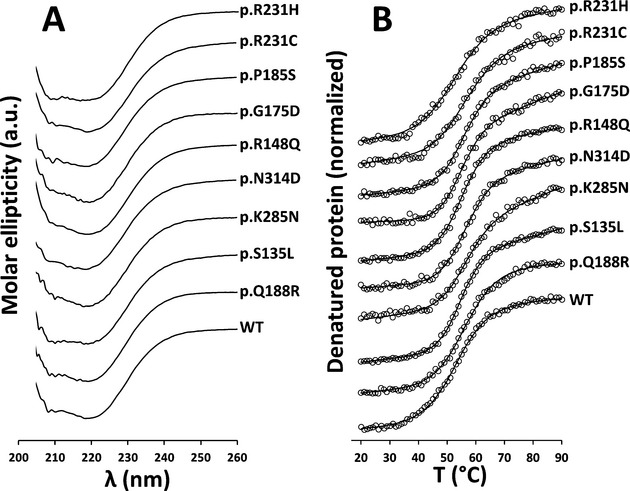
No impact of the studied mutations on human GALT secondary structure. Effect of missense mutations on the secondary structure of GALT variants, probed by Far-UV circular dichroism spectropolarimetry. (A) Far-UV CD spectra, collected for GALT variants, at 20°C, at 0.15 mg/mL, in 50 mmol/L Tris-HCl, 300 mmol/L KCl, 10% glycerol, pH 7.5. Spectra resulted from six accumulations at a 50 nm/min scan rate, in a 0.1 cm light path cuvette. (B) Thermal denaturation profiles obtained by monitoring molar ellipticity at 222 nm in the 20–90°C temperature range (1°C/min slope; data pitch: 1°C; delay time: 0 sec); temperature scan curves were normalized and fitted according to a two-state model (respective *T*_m_ values in Table1). GALT, galactose-1-phosphate uridylyltransferase.

Far-UV CD spectra of all GALT variants were very similar to that of WT, with two minima at 208 and 222 nm (Fig.2), consistent with a combination of *α*-helical and *β*-sheet secondary structure content (six *α*-helices and thirteen *β*-sheets predicted). Thermal denaturation curves, obtained by monitoring the molar ellipticity at 222 nm as a function of constantly increasing temperature, presented an apparently single transition and were fitted according to a two-state model. Thermal denaturation of the GALT variants appeared to be irreversible, since the spectra collected at 20°C after cooling the denatured samples had lost the spectral features assigned to the different secondary structure elements (data not shown). The thermal denaturation profiles of all variants yielded similar *T*_m_ values (Table1), ranging from 52.6 to 56.7°C. With the exception of p.R231H, all variants displayed slightly higher *T*_m_ values than WT GALT, although all the Δ*T*_m_ ± SD fell below the 2°C threshold.

DSF was employed to analyze the effects of the substituting amino acids on tertiary structure elements. The fluorescence intensity measured in the first asymptote of the sigmoidal thermal denaturation profiles (flat over the 20–30°C range) was normalized with respect to the WT values (Fig. S1). As observed, all the variants exhibited similar ground-state fluorescence, the sole exception being the p.Q188R variant, which displayed a value ∼30% higher than that of the WT GALT.

The DSF thermal denaturation profiles for all variants exhibited two apparent transitions, each accounting for 40–60% of the overall fluorescence increase (Fig. S2). The inflexion points of the two transitions, *T*_m1_ and *T*_m2_, fell within a narrow range of temperatures, with *T*_m1_ ranging from 42.0 ± 0.4 to 44.3 ± 0.2°C and *T*_m2_ ranging from 51.0 ± 0.4 to 53.9 ± 0.1°C (Table1). None of the GALT variants exhibited *T*_m_ values ≥2°C higher or lower than those determined for the WT GALT.

The effect of the GALT substrates Gal-1-P and UDP-Glc on the thermal denaturation profiles was tested by DSF. Neither substrate yielded significant changes in either *T*_m_ values (all Δ*T*_m_ <1°C, Table S2). As GALT has two putative mononuclear metal-binding sites, one for iron and another for zinc, DSF assays were carried out in the presence of either metal. The only GALT variant exhibiting a response to Fe^2+^ was p.P185S, its *T*_m1_ increasing by 2.5 ± 0.5°C (Table S2). The presence of Zn^2+^ had two levels of impact on the thermal denaturation profiles and their corresponding *T*_m_ values (Table S2). Whereas the *T*_m1_ values for p.Q188R, p.N314D, and p.R148Q remained unvaried, WT and all other variants exhibited a decrease in *T*_m1_ from −2.5 ± 0.3°C to −5.5 ± 0.3°C. The effect of Zn^2+^ had a deeper impact on the *T*_m2_ values, which decreased significantly (Δ*T*_m2_ between −2.9 ± 0.6°C and −5.2 ± 1.5°C) for the WT GALT and all the variants except p.Q188R.

### Disturbed aggregation of GALT variants

**Figure 3:**
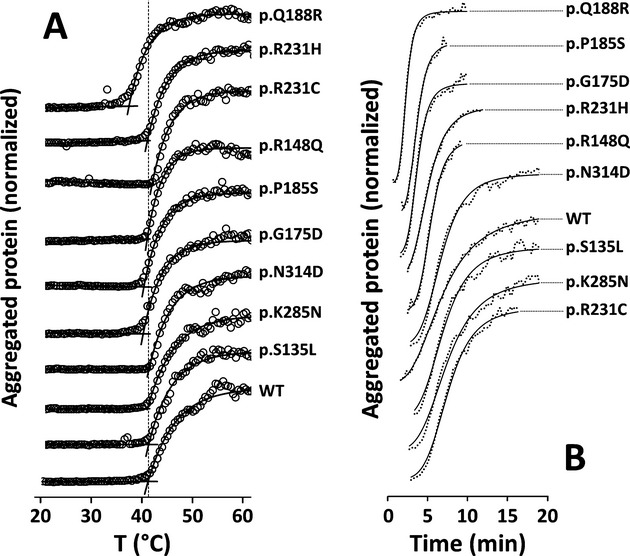
Dynamic light scattering analysis of GALT variants reveals disturbed aggregation. Impact of missense mutations on the aggregation of GALT variants in solution, studied by dynamic light scattering (DLS). All proteins samples were diluted in 50 mmol/L Tris-HCl, 300 mmol/L KCl, 10% glycerol, pH 7.5, to a final concentration of 0.15 mg/mL. (A) Temperature-induced aggregation profiles, obtained by a linear temperature increase from 20°C to 70°C at 0.5°C/min, collecting the particle size average, distribution and total scattering intensity. Scattering intensity data were normalized and fitted to a plateau followed by one phase association equation, the aggregation temperature (*T*_agg_) being defined as the temperature at which the intensity starts to increase significantly. (B) Kinetics of thermal aggregation monitored at 42°C for 60 min. Light scattering intensity is plotted as a function of time, sigmoidal curves were obtained and the *t*_1/2_ was defined as the time elapsed to reach 50% of maximum of aggregation. Asymptotes were removed for clarity, due to the data noise in those regions of the profiles. GALT, galactose-1-phosphate uridylyltransferase.

The propensity of GALT variants to aggregate in solution was analyzed by DLS, evaluating the *T*_agg_ and also the aggregation kinetics at two different temperatures (37 and 42°C). Scanning the particle size as a function of temperature, the estimated *T*_agg_ were essentially identical for all GALT variants herein studied (ranging from 40.1 ± 1.0 to 41.4 ± 0.1°C), except the p.Q188R variant, which started to aggregate at a lower temperature (Δ*T*_agg_ of −3.9°C, with respect to the WT GALT) (Fig.3A and Table1).

The aggregation kinetics was monitored by determining the *t*_1/2_ of aggregation at 37°C and 42°C, representing, respectively, a physiological body temperature and a thermal insult. At 37°C, whereas the p.K285N, p.R148Q, and p.R231H variants displayed similar *t*_1/2_ as the WT GALT (∼27 min), the other variants exhibited disturbed agg-regation profiles, aggregating either faster (p.Q188R, p.G175D, and p.P185S, approximate Δ*t*_1/2_ respectively −20, −12, and −16 min) or slower (p.S135L, p.N314D, and p.R231C, approximate Δ*t*_1/2_ respectively +10, +15, and +22 min) than the WT GALT (Fig. S3 and Table1). At 42°C, the aggregation was sped-up, lowering the *t*_1/2_ of all GALT variants, which aggregate as fast as the WT GALT (*t*_1/2_ ∼ 7 min; p.S135L, p.K285N, p.N314D, and p.R231C) or faster (*t*_1/2_ between 2.3 and 5 min; p.Q188R, p.R148Q, p.G175D, p.P185S, and p.R231H) (Fig.2B and Table1).

## Discussion

The mutational spectrum of classic galactosemia is dominated by missense mutations. As the current standard of care based on a galactose-restricted diet fails to prevent long-term complications, a deeper knowledge on the molecular basis of *GALT* mutations pathogenicity will support the design of new therapeutic strategies. We report the structural–functional characterization of nine clinically relevant GALT variants, four of which result from the most prevalent *GALT* mutant alleles: p.Q188R, p.S135L, p.K285N, and p.N314D.

Recombinant WT GALT displayed an enzymatic activity and kinetic parameters toward Gal-1-P and UDP-Glc compatible with reported values obtained by a direct UDP-Gal quantitation HPLC assay (Lindhout et al. 2010). Only four GALT variants displayed activity above the assay's detection limit (p.Q188R, p.S135L, p.N314D, and p.G175D), even using a fivefold higher protein concentration than WT GALT. Whereas p.N314D had the same specific activity as WT GALT, the other variants displayed ≤0.2% of residual activity. Previous studies reported undetectable to 0.7% of WT activity for recombinant p.Q188R, produced in yeast (Fridovich-Keil and Jinks-Robertson 1993) or bacteria (Lai et al. 1999); and undetectable to 5% of WT activity for p.S135L, determined in homozygous patients' cells (Lai et al. 1996) or in yeast lysates expressing the recombinant variant (Wells and Fridovich-Keil 1997). Thermal inactivation profiles showed the variants with detectable enzymatic activity were more sensitive than WT GALT to thermal inactivation, indicating an impaired functional and/or conformational stability (Table1).

Far-UV CD spectropolarimetry was used to probe the impact of the studied mutations on the secondary structure elements of GALT variants, which displayed overlapping CD spectral features with the WT protein (Fig.2), showing that the studied mutations have no significant effects on the variants' secondary structure topology, as previously reported for *E. coli* GalT and its p.Q168R variant, equivalent to human p.Q188R (Geeganage and Frey 1998). Structural models obtained for each variant (Figs. S4–S12) indicate that the substituting residues have limited or null effects on secondary structure elements. Thermal denaturation profiles (Fig.2) confirmed that WT GALT and all variants displayed very similar secondary structure thermal stability, with |Δ*T*_m_| ± S.D. <2°C (Table1).

Impact of the mutations on the tertiary structure of GALT variants was evaluated by DSF. Whereas most variants exhibited a “ground-state” fluorescence similar to WT GALT, a >30% increase was observed for p.Q188R (Fig. S1), indicating that the p.Q188R native conformation displays a higher and/or more prolonged exposure of hydrophobic residues. DSF thermal denaturation curves showed two transitions (Fig. S2), suggestive of two protein regions unfolding as separate domains, contrarily to the previously reported single transition for different GALT variants (McCorvie et al. 2013). Both *T*_m_ values determined for the WT GALT (43.7 ± 0.7°C and 52.4 ± 1.2°C) are lower than the previously reported *T*_m_ (63°C) (McCorvie et al. 2013), which could be partially explained by different experimental conditions, particularly a pH closer to physiologic in our assays (7.5 vs. 8.8 reported in (McCorvie et al. 2013)), besides the protein concentrations and the temperature slope (McCorvie et al. 2013). Concerning the variants, the *T*_m_ values for each transition displayed no significant differences relative to WT GALT (Table1), as all |Δ*T*_m_| < 2°C, ruling out any significant effect of the mutations on the tertiary structure thermal stability.

DSF assays were also employed to evaluate the effect of GALT substrates (Gal-1-P and UDP-Glc) on the GALT variants' conformational stability (Table S2). We observed no effect of either substrate on the *T*_m_ values for WT GALT and the studied variants, whereas McCorvie et al. previously reported a stabilizing effect of both substrates for WT GALT and the p.D28Y and p.F194L variants (McCorvie et al. 2013). Notably, the structures of the *E. coli* GalT in the native and nucleotylated states (PDB ID 1HXP and 1HXQ) are totally overlapping (Wedekind et al. 1995, 1996), ruling out major conformational changes upon substrate binding.

Zinc and iron were shown to have a structural role in bacterial GalT (Ruzicka et al. 1995). While the iron-binding ligands are fully conserved in human GALT, the zinc ligands are partially conserved, raising the question whether this metal is essential (Wells and Fridovich-Keil 1997). While iron had no effect on the WT GALT stability in the DSF assays, zinc had the puzzling effect of destabilizing the protein, lowering both *T*_m_ values by ∼4°C (Table S2). As the zinc binding pocket is not fully conserved, our results suggest that zinc may occupy the mononuclear iron-binding site, proposed to be unable to completely discriminate between iron and zinc (Geeganage and Frey 1999; Holden et al. 2003). Zinc may also bind to the exposed N-terminal 6-His tag and partially affect protein stability (Evers et al. 2008). Iron had no significant effects on any variant except for a slight increase in the *T*_m1_ of p.P185S, located far from the iron-binding site, suggesting that local perturbations propagate to distal sites in the protein structure. Zinc, however, resulted in all variants behaving essentially as the WT GALT (Table S2) except p.Q188R, which appeared insensitive to zinc. The replaced serine in p.S135L structurally overlaps with the zinc-binding ligand H115 in *E. coli* GalT. The fact that this variant displays the same zinc sensitivity as WT GALT reinforces the idea that the observed zinc-induced destabilization might be related to non-specific occupation of the iron-binding site. Although the effect of zinc cannot as yet be rationalized in functional terms, the different impacts on the conformational stability suggest subtle structural differences between these variants.

As aggregation in solution is a hallmark of protein misfolding, DLS was used to compare the proneness of the GALT variants to aggregate, evaluating the *T*_agg_ and the aggregation kinetics at 37°C and upon a thermal insult at 42°C (Fig.3 and Table1). Regarding the thermal aggregation profiles, all variants but p.Q188R exhibited *T*_agg_ values nearly identical to the WT GALT. p.Q188R displayed a disturbed thermal aggregation profile, with a *T*_agg_ ∼4°C lower than WT, indicating a higher propensity to aggregate in solution. Recalling the ∼30% higher ground-state fluorescence of this variant as compared to WT GALT, we hypothesize that a higher and/or longer exposure of hydrophobic residues may be directing its increased tendency to aggregate. The aggregation kinetics at two different temperatures also highlighted the disturbed aggregation behavior of other variants. At 37°C, the p.G175D and p.P185S variants also aggregate significantly faster than WT GALT, whereas p.S135L, p.N314D and p.R231C displayed slower aggregation kinetics. Upon thermal insult at 42°C, some of the effects observed at 37°C were enhanced, others leveled out, and new effects were observed. Besides p.Q188R, p.G175D and p.P185S, also p.R148Q and p.R231H aggregate faster than WT GALT at 42°C, indicating that the latter two variants are less resistant to aggregation under this thermal insult. All the variants that presented slower aggregation at 37°C were leveled to the WT aggregation kinetics at 42°C. p.K285N was the only variant showing aggregation parameters essentially identical to the WT GALT in both conditions.

The results from the different methodologies herein employed indicate that the major structural impact of the studied mutations concerns the aggregation in solution, with no significant effects on the secondary and tertiary structures. To support our understanding of the functional and structural impairment of GALT variants, we generated structural models of each one (Figs.1C and S4–S12). p.Q188R has generally been regarded as a functional variant, since in the bacterial structure the substituted glutamine establishes through its amide moiety two H-bonds towards UDP-Gal (Geeganage and Frey 1998). In the bacterial p.Q168R – equivalent to human p.Q188R – one of these H-bonds is absent. This variant has also been proposed to be affected in inter-subunit interactions (Marabotti and Facchiano 2005; Facchiano and Marabotti 2010). The p.Q188R model (Figs.1C and S4) suggests an actual gain in H-bonds, since the guanidinium moiety may establish three “new” H-bonds towards the intermediate phosphate and sugar moieties. This variant's functional impairment could therefore result from over-stabilization of the substrates and/or products blocking the enzyme active site for further reaction turnover. Such a disturbed intermediate stabilization had already been proposed by Marabotti and Facchiano (2005), who remarked that this analysis is highly dependent on the accuracy of the predicted location of the R188 side-chain, observed to adopt different geometries among different models (Fig.1C). In addition to the local H-bond network differences, we observed a significant change in the electrostatic surface surrounding this position (Fig. S4), consistent with the substitution of a globally neutral amide with the positively charged guanidinium of arginine, which may also affect binding of mostly negatively charged reaction substrates and products. It remains to be clarified how this substitution renders this variant insensitive to zinc-induced destabilization. Taken together, these observations demonstrate how local subtle changes can propagate into other regions of the protein with dramatic global effects.

Besides local changes in the H-bonds and electrostatic interactions resulting from the studied mutations, most studied variants that presented disturbed aggregation kinetics also displayed predicted effects on their surface electrostatic charges, either inverting the polarity of the local charges, or neutralizing them (Figs. S4–S12). Although this is not a necessary condition to alter the aggregation propensity, it may contribute to stabilize non-native conformations with downstream effects on protein aggregation. Moreover, taking into account the proposal that GALT may assemble with GALE and GALK a supra-molecular structure localized to discrete spots in the cell (Christacos et al. 2000; McCorvie and Timson 2011), surface charge perturbations could hamper the formation of such complexes. Since the structural and molecular modeling studies do not unequivocally explain the disturbed aggregation profiles, it is likely that the subtle structural changes resulting from each mutation have an impact on the protein dynamics, extending the lifetime of conformations with a higher exposure of hydrophobic residues, that could act as nuclei triggering aggregation of the misfolded variants (e.g., Saunders and Bottomley 2009; Kubota et al. 2011).

Several studies have aimed to analyze the functional impairment of the most prevalent mutations in classic galactosemia. A recent report suggests protein misfolding as the pathogenic mechanism of several *GALT* missense mutations, as commonly observed for genetic diseases (Gregersen et al. 2006; McCorvie et al. 2013). In our functional and structural impact studies on the most frequent variations in classic galactosemia (p.Q188R, p.S135L, p.K285N, and p.N314D) accounting for the vast majority of mutant alleles at a global scale, the most striking and novel observation is that most variants display disturbed aggregation profiles, despite the absence of detectable structural effects on their secondary and tertiary structures. This is particularly relevant for p.Q188R, resulting from the most prevalent mutation, accounting for ∼60% of the mutant alleles. This observation is extremely important, since at the cellular level, the accumulation of aggregation-prone proteins interferes severely with the cellular homeostasis. Fibroblasts from p.Q188R homozygous patients displayed increased endoplasmic reticulum stress (Slepak et al. 2007). Moreover, studies on GALT-null galactosemia models revealed increased unfolded protein response (De-Souza et al. 2014) and oxidative stress levels (Jumbo-Lucioni et al. 2013), also suggesting that there is a basal level of protein homeostasis disturbance associated with galactosemia. There is an increasing awareness that accumulation of damaged or abnormal proteins is the underlying pathogenic molecular mechanism of several diseases, and several studies on inborn errors of metabolism revealed protein aggregation as a more common pathogenic mechanism than previously thought (Pedersen et al. 2003). A relevant example concerns the in vivo GALE aggregation in type III galactosemia (Bang et al. 2009). Accordingly, the results from the structural analyses of the GALT mutants herein characterized strongly suggest that GALT aggregation associated with protein misfolding might be a major pathogenic mechanism in classic galactosemia, setting the basis for future studies on in vivo GALT aggregation. Therefore, an intervention at the level of proteostasis modulation and correction of protein misfolding (by chemical and/or pharmacological chaperones) could not only increase the lifetime of partially active variants, but also prevent the accumulation of protein aggregates and simultaneously alleviate the disease phenotype associated with protein homeostasis disturbances.

## Supporting Information

**Figure S1.** “Ground-state” extrinsic fluorescence of GALT variants in the presence of fluorescent dye targeting hydrophobic regions. Thermal denaturation profiles were obtained by differential scanning fluorimetry assays, probing the impact of mutations on the tertiary structure of GALT variants. “Ground-state” extrinsic fluorescence was estimated as the averaged fluorescence intensities recorded in the first asymptote (20–30°C) of the sigmoidal thermal denaturation profiles. Histogram represents “ground-state” fluorescence of GALT variants normalized for the wild-type GALT values. Dashed line represents the wild-type GALT level. **Figure S2.** The studied mutations have a limited impact on the tertiary structure of GALT variants. Thermal denaturation profiles were obtained by differential scanning fluorimetry assays, probing the impact of somatic mutations on the tertiary structure of GALT variants. The reaction mixture, totaling 50 *μ*L, contained 0.1 mg/mL protein (∼2.5 μmol/L in monomer) in 50 mmol/L Tris-HCl, 300 mmol/L KCl, 10% glycerol, pH 7.5, and 5× SYPRO Orange. After a 10-min incubation at 20°C, temperature was linearly increased from 20 to 90°C at 1°C/min, with HEX channel fluorescence acquisition every 0.2°C. Temperature scan curves were averaged, normalized and fitted to a biphasic dose-response. *T*_m_ values were estimated from the inflexion points of the first and second transitions (Table1). **Figure S3.** Thermal aggregation kinetics probed by dynamic light scattering. Kinetics of thermal aggregation monitored at 37°C for 60 min. Light scattering intensity are plotted as a function of time, sigmoidal curves were obtained and the *t*_1/2_ was defined as the time elapsed to reach 50% of maximum of aggregation. Asymptotes were removed for clarity, due to the data noise in those regions of the profiles.

**Figure S4.** Structural impact of the p.Q188R substitution in human GALT. Structural model of human p.Q188R GALT (grey cartoon) and opposing monomer from *Escherichia coli* GalT (orange ribbon, PDB ID: 1GUP). *Top panel*, Q188 is represented in grey and the substituting R188 is in red; light blue cartoon highlights the location of the active site H_184_-P_185_-H_186_ active site residues. *Bottom panels*, surface electrostatics map surrounding the Q188 residue (*left*) and the substituting R188 residue (*right*); light blue sticks, bound UDP-glucose; white dotted circles highlight the Q188/R188 location. Red, negative charge; blue, positive charge; white, neutral. Figure generated with PyMOL. **Figure S5.** Structural impact of the p.S135L substitution in human GALT. Structural model of human p.S135L GALT (grey cartoon) and opposing monomer from *Escherichia coli* GalT (orange ribbon, PDB code 1GUP). S135 is represented in grey and the substituting L135 is in red; light blue cartoon highlights the location of the active site H_184_-P_185_-H_186_ active site residues; purple sphere, Zn^2+^ ion from 1GUP PDB. Figure generated with PyMOL. **Figure S6.** Structural impact of the p.K285N substitution in human GALT. Structural model of human p.K285N GALT (grey cartoon) and opposing monomer from *Escherichia coli* GalT (orange ribbon, PDB code 1GUP). *Top panel*, K285 is represented in grey and the substituting N285 is in red; light blue cartoon highlights the location of the active site H_184_-P_185_–H_186_ active site residues. *Bottom panels*, surface electrostatics map surrounding the K285 residue (*left*) and the substituting N285 residue (*right*); white dotted circles highlight the K285/N285 location. Red, negative charge; blue, positive charge; white, neutral. Figure generated with PyMOL. **Figure S7.** Structural impact of the p.N314D substitution in human GALT. Structural model of human p.N314D GALT (grey cartoon) and opposing monomer from *Escherichia coli* GalT (orange ribbon, PDB code 1GUP). *Top panel*, N314 is represented in grey and the substituting D314 is in red; orange spheres, Fe^2+^ ions from 1GUP PDB. *Bottom panels*, surface electrostatics map surrounding the N314 residue (*left*) and the substituting D314 residue (*right*); white dotted circles highlight the N314/D314 location. Red, negative charge; blue, positive charge; white, neutral. Figure generated with PyMOL. **Figure S8.** Structural impact of the p.R148Q substitution in human GALT. Structural model of human p.R148Q GALT (grey cartoon) and opposing monomer from *Escherichia coli* GalT (orange ribbon, PDB code 1GUP). *Top panel*, R148 is represented in grey and the substituting Q148 is in red. *Bottom panels*, surface electrostatics map surrounding the R148 residue (*left*) and the substituting Q148 residue (*right*); white dotted circles highlight the R148/Q148 location. Red, negative charge; blue, positive charge; white, neutral. Figure generated with PyMOL. **Figure S9.** Structural impact of the p.G175D substitution in human GALT. Structural model of human p.G175D GALT (grey cartoon) and opposing monomer from *Escherichia coli* GalT (orange ribbon, PDB code 1GUP). *Top panel*, G175 is represented in grey and the substituting D175 is in red; orange sphere, Fe^2+^ ion from 1GUP PDB. *Bottom panels*, surface electrostatics map surrounding the G175 residue (*left*) and the substituting D175 residue (*right*); white dotted circles highlight the G175/D175 location. Red, negative charge; blue, positive charge; white, neutral. Figure generated with PyMOL. **Figure S10.** Structural impact of the p.P185S substitution in human GALT. Structural model of human p.P185S GALT (grey cartoon) and opposing monomer from *Escherichia coli* GalT (orange ribbon, PDB code 1GUP). P185 is represented in grey and the substituting S185 is in red; light blue cartoon highlights the location of the active site H_184_-P_185_-H_186_ active site residues; purple and orange sphere, respectively, Zn^2+^ and Fe^2+^ ions from 1GUP PDB. Figure generated with PyMOL. **Figure S11.** Structural impact of the p.R231C substitution in human GALT. Structural model of human p.R231C GALT (grey cartoon) and opposing monomer from *Escherichia coli* GalT (orange ribbon, PDB code 1GUP). *Top panel*, R231 is represented in grey and the substituting C231 is in red. *Bottom panels*, surface electrostatics map surrounding the R231 residue (*left*) and the substituting C231 residue (*right*); white dotted circles highlight the R231/C231 location. Red, negative charge; blue, positive charge; white, neutral. Figure generated with PyMOL. **Figure S12.** Structural impact of the p.R231H substitution in human GALT. Structural model of human p.R231H GALT (grey cartoon) and opposing monomer from *Escherichia coli* GalT (orange ribbon, PDB code 1GUP). *Top panel*, R231 is represented in grey and the substituting H231 is in red. *Bottom panels*, surface electrostatics map surrounding the R231 residue (*left*) and the substituting H231 residue (*right*); white dotted circles highlight the R231/H231 location. Red, negative charge; blue, positive charge; white, neutral. Figure generated with PyMOL.

Table S1 Oligonucleotides used for site-directed mutagenesis.

Table S2 Effect of GALT substrates and putative metal cofactors on the conformational stability of GALT variants. Thermal shifts observed on WT and variant GALT proteins induced by addition of the substrates galactose-1-phosphate (Gal-1-P) and UDP-glucose (UDP-Glc) and the metal ions (Fe^2+^ and Zn^2+^), monitored by differential scanning fluorimetry.
