# Supplementary material for: Functional and structural impact of the most prevalent missense mutations in classic galactosemia
Source: Mol Genet Genomic Med. 2014 Jun 23;2(6):484–96. doi: 10.1002/mgg3.94 (PMC4303218; doi:10.1002/mgg3.94)
Supplement: Supplementary file 1 — Figure S1. “Ground-state” extrinsic fluorescence of GALT variants in the presence of fluorescent dye targeting hydrophobic regions. Thermal denaturation profiles were obtained by differential scanning fluorimetry assays, probing the impact of mutations on the tertiary structure of GALT variants. “Ground-state” extrinsic fluorescence was estimated as the averaged fluorescence intensities recorded in the first asymptote (20–30°C) of the sigmoidal thermal denaturation profiles. Histogram represents “ground-state” fluorescence of GALT variants normalized for the wild-type GALT values. Dashed line represents the wild-type GALT level. Figure S2. The studied mutations have a limited impact on the tertiary structure of GALT variants. Thermal denaturation profiles were obtained by differential scanning fluorimetry assays, probing the impact of somatic mutations on the tertiary structure of GALT variants. The reaction mixture, totaling 50 μL, contained 0.1 mg/mL protein (∼2.5 μmol/L in monomer) in 50 mmol/L Tris-HCl, 300 mmol/L KCl, 10% glycerol, pH 7.5, and 5× SYPRO Orange. After a 10-min incubation at 20°C, temperature was linearly increased from 20 to 90°C at 1°C/min, with HEX channel fluorescence acquisition every 0.2°C. Temperature scan curves were averaged, normalized and fitted to a biphasic dose-response. Tm values were estimated from the inflexion points of the first and second transitions (Table1). Figure S3. Thermal aggregation kinetics probed by dynamic light scattering. Kinetics of thermal aggregation monitored at 37°C for 60 min. Light scattering intensity are plotted as a function of time, sigmoidal curves were obtained and the t1/2 was defined as the time elapsed to reach 50% of maximum of aggregation. Asymptotes were removed for clarity, due to the data noise in those regions of the profiles. [file mgg30002-0484-sd1.docx]

| **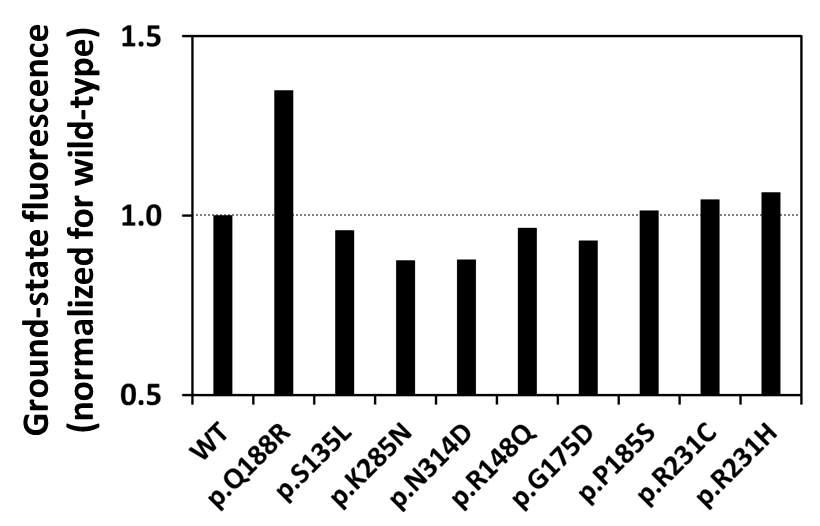** |
| --- |
| **Figure S1 - ‘Ground-state’ extrinsic fluorescence of GALT variants in the presence of fluorescent dye targeting hydrophobic regions.** Thermal denaturation profiles were obtained by differential scanning fluorimetry assays, probing the impact of mutations on the tertiary structure of GALT variants. ‘Ground-state’ extrinsic fluorescence was estimated as the averaged fluorescence intensities recorded in the first asymptote (20-30 °C) of the sigmoidal thermal denaturation profiles. Histogram represents ‘ground-state’ fluorescence of GALT variants normalized for the wild-type GALT values. Dashed line represents the wild-type GALT level. |

**Figure S1 Coelho (2014)**

| 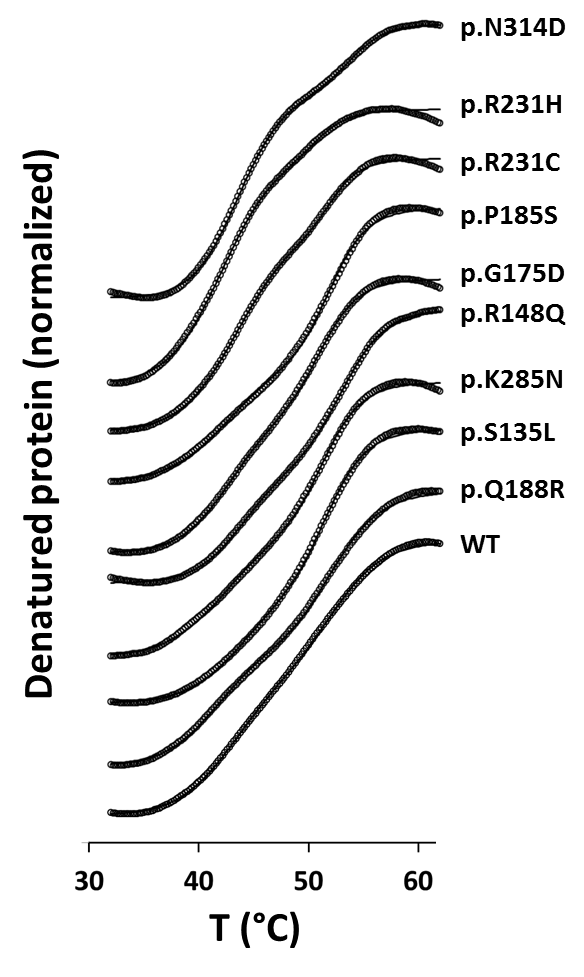 |
| --- |
| **Figure S2 -** The studied mutations have a limited impact on the tertiary structure of GALT variants. Thermal denaturation profiles were obtained by differential scanning fluorimetry assays, probing the impact of mutations on the tertiary structure of GALT variants. The reaction mixture, totaling 50 μl, contained 0.1 mg/mL protein (~2.5 μM in monomer) in 50 mM Tris-HCl, 300 mM KCl, 10 % glycerol, pH 7.5, and 5x SYPRO Orange. After a 10-min incubation at 20 °C, temperature was linearly increased from 20 to 90 °C at 1 °C/min, with HEX channel fluorescence acquisition every 0.2 °C. Temperature scan curves were averaged, normalized and fitted to a biphasic dose-response. *T*_m_ values were estimated from the inflexion points of the first and second transitions (Table 1). |

**Figure S2 Coelho (2014)**

| **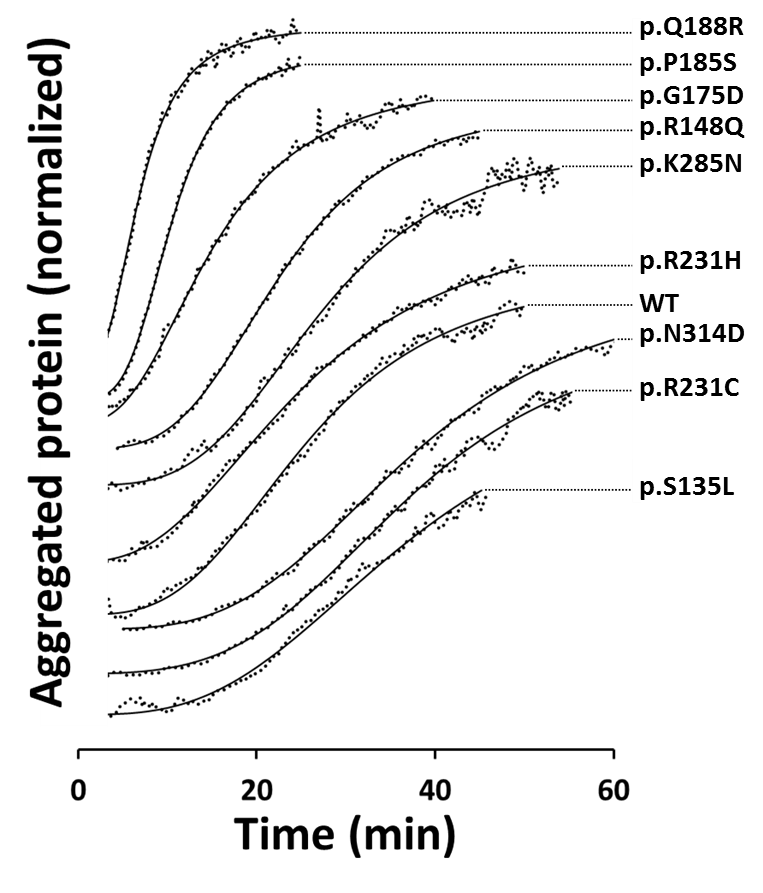** |
| --- |
| **Figure S3 - Thermal aggregation kinetics probed by dynamic light scattering.** Kinetics of thermal aggregation monitored at 37 °C for 60 min. Light scattering intensity are plotted as a function of time, sigmoidal curves were obtained and the t_1/2_ was defined as the time elapsed to reach 50% of maximum of aggregation. Asymptotes were removed for clarity, due to the data noise in those regions of the profiles. |

**Figure S3 Coelho (2014)**
