# Supplementary material for: Functional and structural impact of the most prevalent missense mutations in classic galactosemia
Source: Mol Genet Genomic Med. 2014 Jun 23;2(6):484–96. doi: 10.1002/mgg3.94 (PMC4303218; doi:10.1002/mgg3.94)
Supplement: Supplementary file 2 — Figure S4. Structural impact of the p.Q188R substitution in human GALT. Structural model of human p.Q188R GALT (grey cartoon) and opposing monomer from Escherichia coli GalT (orange ribbon, PDB ID: 1GUP). Top panel, Q188 is represented in grey and the substituting R188 is in red; light blue cartoon highlights the location of the active site H184-P185-H186 active site residues. Bottom panels, surface electrostatics map surrounding the Q188 residue (left) and the substituting R188 residue (right); light blue sticks, bound UDP-glucose; white dotted circles highlight the Q188/R188 location. Red, negative charge; blue, positive charge; white, neutral. Figure generated with PyMOL. Figure S5. Structural impact of the p.S135L substitution in human GALT. Structural model of human p.S135L GALT (grey cartoon) and opposing monomer from Escherichia coli GalT (orange ribbon, PDB code 1GUP). S135 is represented in grey and the substituting L135 is in red; light blue cartoon highlights the location of the active site H184-P185-H186 active site residues; purple sphere, Zn2+ ion from 1GUP PDB. Figure generated with PyMOL. Figure S6. Structural impact of the p.K285N substitution in human GALT. Structural model of human p.K285N GALT (grey cartoon) and opposing monomer from Escherichia coli GalT (orange ribbon, PDB code 1GUP). Top panel, K285 is represented in grey and the substituting N285 is in red; light blue cartoon highlights the location of the active site H184-P185–H186 active site residues. Bottom panels, surface electrostatics map surrounding the K285 residue (left) and the substituting N285 residue (right); white dotted circles highlight the K285/N285 location. Red, negative charge; blue, positive charge; white, neutral. Figure generated with PyMOL. Figure S7. Structural impact of the p.N314D substitution in human GALT. Structural model of human p.N314D GALT (grey cartoon) and opposing monomer from Escherichia coli GalT (orange ribbon, PDB code 1GUP). Top panel, N314 is repres [file mgg30002-0484-sd2.docx]

| 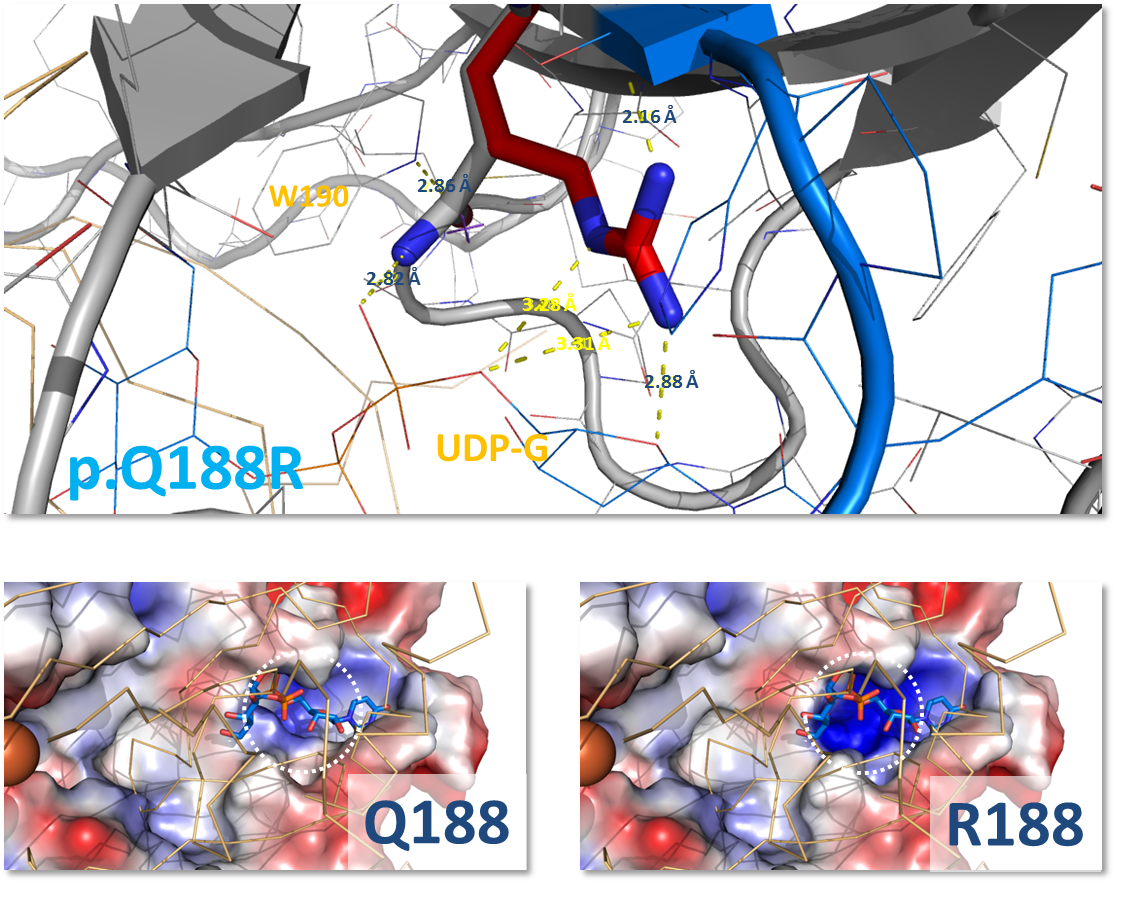 |
| --- |
| **Figure S4 -** Structural impact of the p.Q188R mutation in human GALT. Structural model of human p.Q188R GALT (grey cartoon) and opposing monomer from *Escherichia coli* GalT (orange ribbon, PDB ID: 1GUP). *Top panel*, Q188 is represented in grey and the substituting R188 is in red; light blue cartoon highlights the location of the active site H_184_-P_185_-H_186_ active site residues. *Bottom panels*, surface electrostatics map surrounding the Q188 residue (*left*) and the substituting R188 residue (*right*); light blue sticks, bound UDP-glucose; white dotted circles highlight the Q188/R188 location. Red, negative charge; blue, positive charge; white, neutral. Figure generated with PyMOL. |

**Figure S4 Coelho (2014)**

| 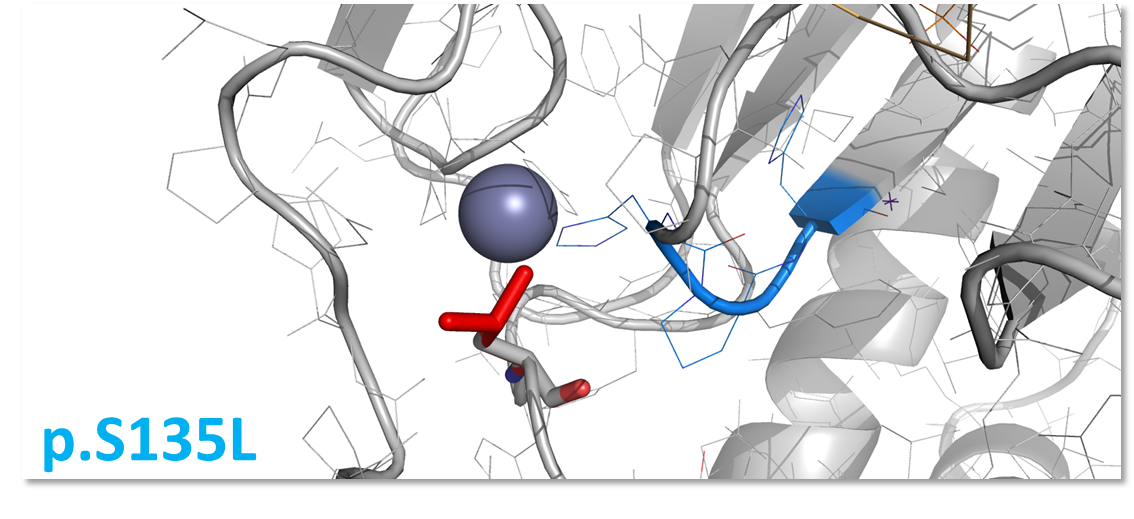 |
| --- |
| **Figure S5 -** Structural impact of the p.S135L mutation in human GALT. Structural model of human p.S135L GALT (grey cartoon) and opposing monomer from *Escherichia coli* GalT (orange ribbon, PDB code 1GUP). S135 is represented in grey and the substituting L135 is in red; light blue cartoon highlights the location of the active site H_184_-P_185_-H_186_ active site residues; purple sphere, Zn^2+^ ion from 1GUP PDB. Figure generated with PyMOL. |

**Figure S5 Coelho (2014)**

| 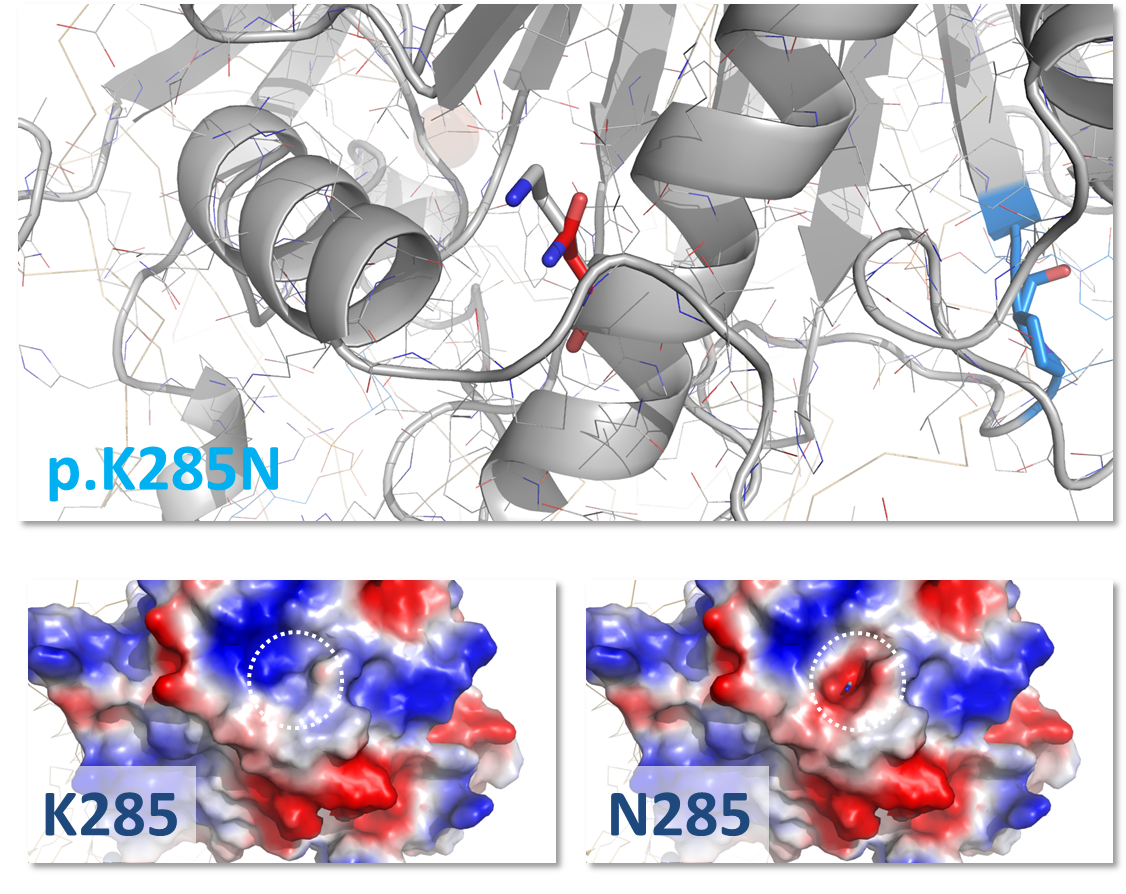 |
| --- |
| **Figure S6 -** Structural impact of the p.K285N mutation in human GALT. Structural model of human p.K285N GALT (grey cartoon) and opposing monomer from *Escherichia coli* GalT (orange ribbon, PDB code 1GUP). *Top panel*, K285 is represented in grey and the substituting N285 is in red; light blue cartoon highlights the location of the active site H_184_-P_185_-H_186_ active site residues. *Bottom panels*, surface electrostatics map surrounding the K285 residue (*left*) and the substituting N285 residue (*right*); white dotted circles highlight the K285/N285 location. Red, negative charge; blue, positive charge; white, neutral. Figure generated with PyMOL. |

**Figure S6 Coelho (2014)**

| 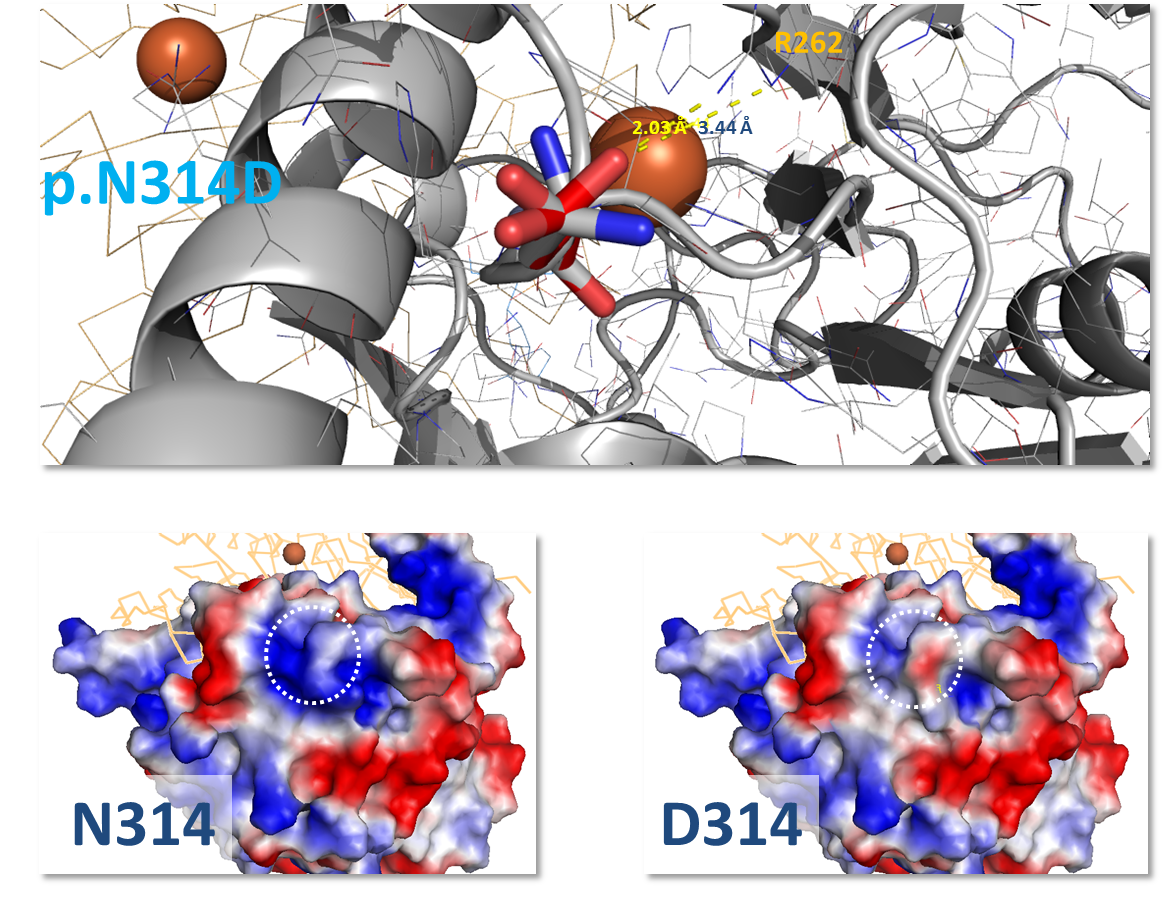 |
| --- |
| **Figure S7 -** Structural impact of the p.N314D mutation in human GALT. Structural model of human p.N314D GALT (grey cartoon) and opposing monomer from *Escherichia coli* GalT (orange ribbon, PDB code 1GUP). *Top panel*, N314 is represented in grey and the substituting D314 is in red; orange spheres, Fe^2+^ ions from 1GUP PDB. *Bottom panels*, surface electrostatics map surrounding the N314 residue (*left*) and the substituting D314 residue (*right*); white dotted circles highlight the N314/D314 location. Red, negative charge; blue, positive charge; white, neutral. Figure generated with PyMOL |

**Figure S7 Coelho (2014)**

| 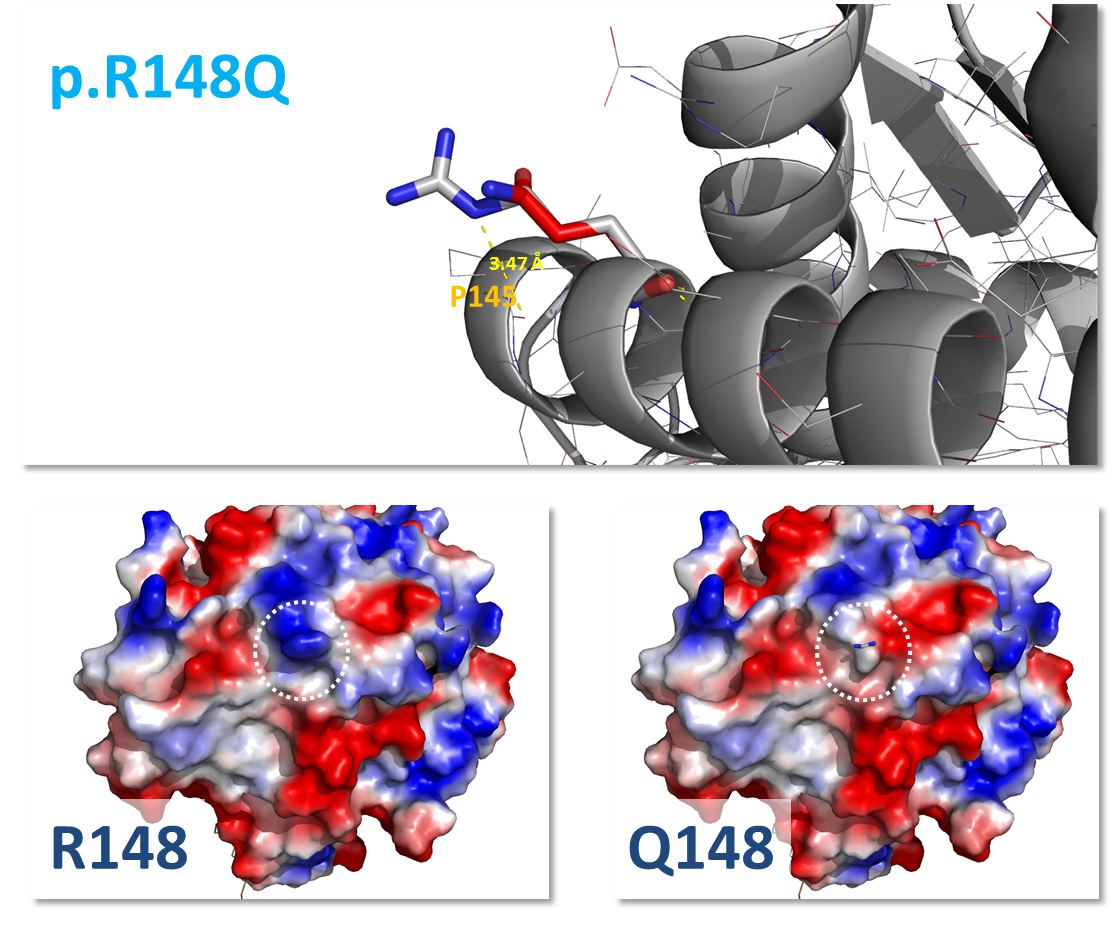 |
| --- |
| **Figure S8** **-** **Structural impact of the p.R148Q mutation in human GALT**. Structural model of human p.R148Q GALT (grey cartoon) and opposing monomer from *Escherichia coli* GalT (orange ribbon, PDB code 1GUP). *Top panel*, R148 is represented in grey and the substituting Q148 is in red. *Bottom panels*, surface electrostatics map surrounding the R148 residue (*left*) and the substituting Q148 residue (*right*); white dotted circles highlight the R148/Q148 location. Red, negative charge; blue, positive charge; white, neutral. Figure generated with PyMOL. |

**Figure S8 Coelho (2014)**

| 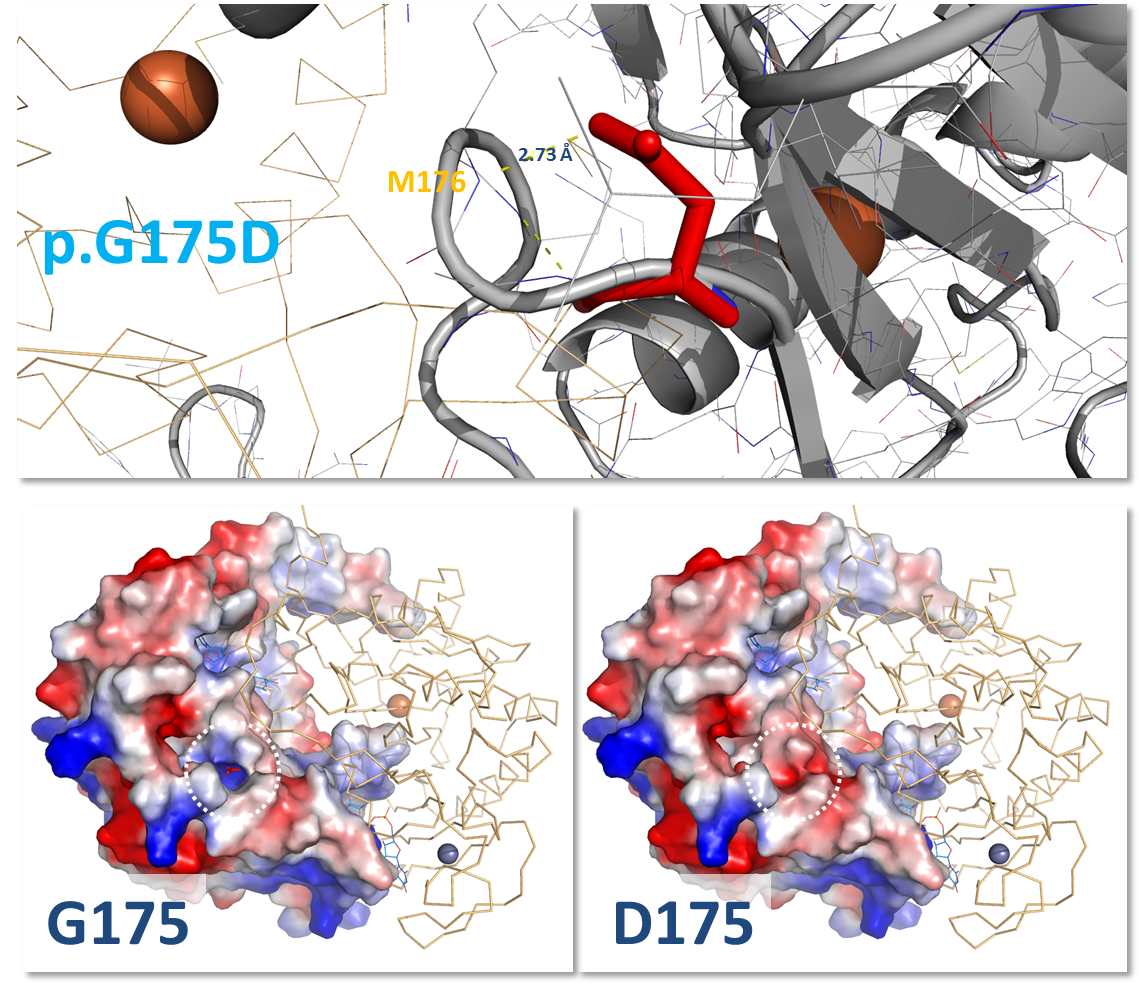 |
| --- |
| **Figure S9 -** Structural impact of the p.G175D mutation in human GALT. Structural model of human p.G175D GALT (grey cartoon) and opposing monomer from *Escherichia coli* GalT (orange ribbon, PDB code 1GUP). *Top panel*, G175 is represented in grey and the substituting D175 is in red; orange sphere, Fe^2+^ ion from 1GUP PDB. *Bottom panels*, surface electrostatics map surrounding the G175 residue (*left*) and the substituting D175 residue (*right*); white dotted circles highlight the G175/D175 location. Red, negative charge; blue, positive charge; white, neutral. Figure generated with PyMOL. |

**Figure S9 Coelho (2014)**

| 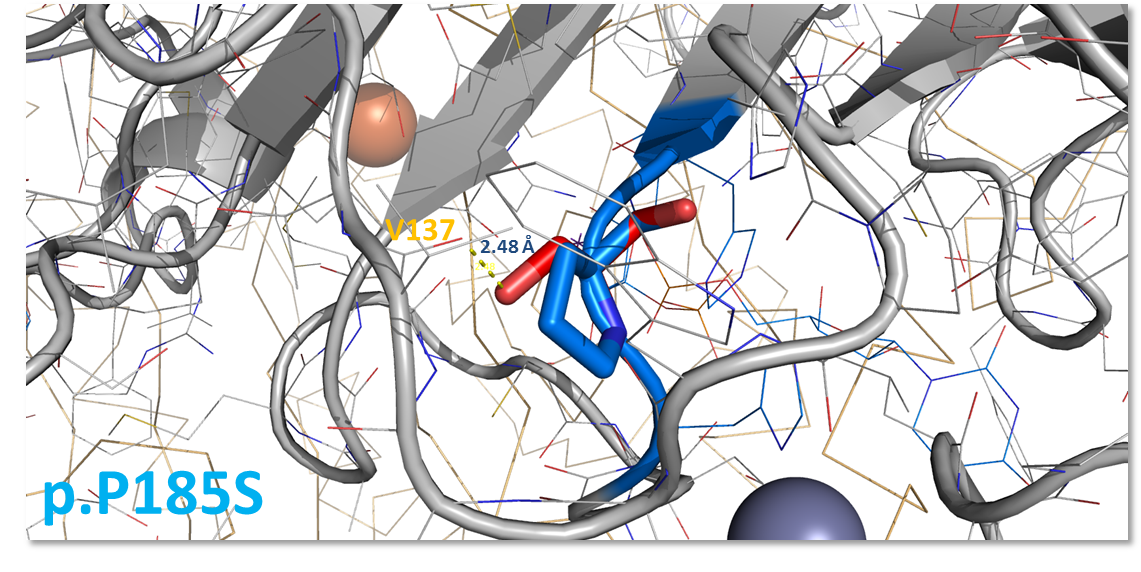 |
| --- |
| **Figure S10 -** Structural impact of the p.P185S mutation in human GALT. Structural model of human p.P185S GALT (grey cartoon) and opposing monomer from *Escherichia coli* GalT (orange ribbon, PDB code 1GUP). P185 is represented in grey and the substituting S185 is in red; light blue cartoon highlights the location of the active site H_184_-P_185_-H_186_ active site residues; purple and orange sphere, respectively Zn^2+^ and Fe^2+^ ions from 1GUP PDB. Figure generated with PyMOL. |

**Figure S10 Coelho (2014)**

| 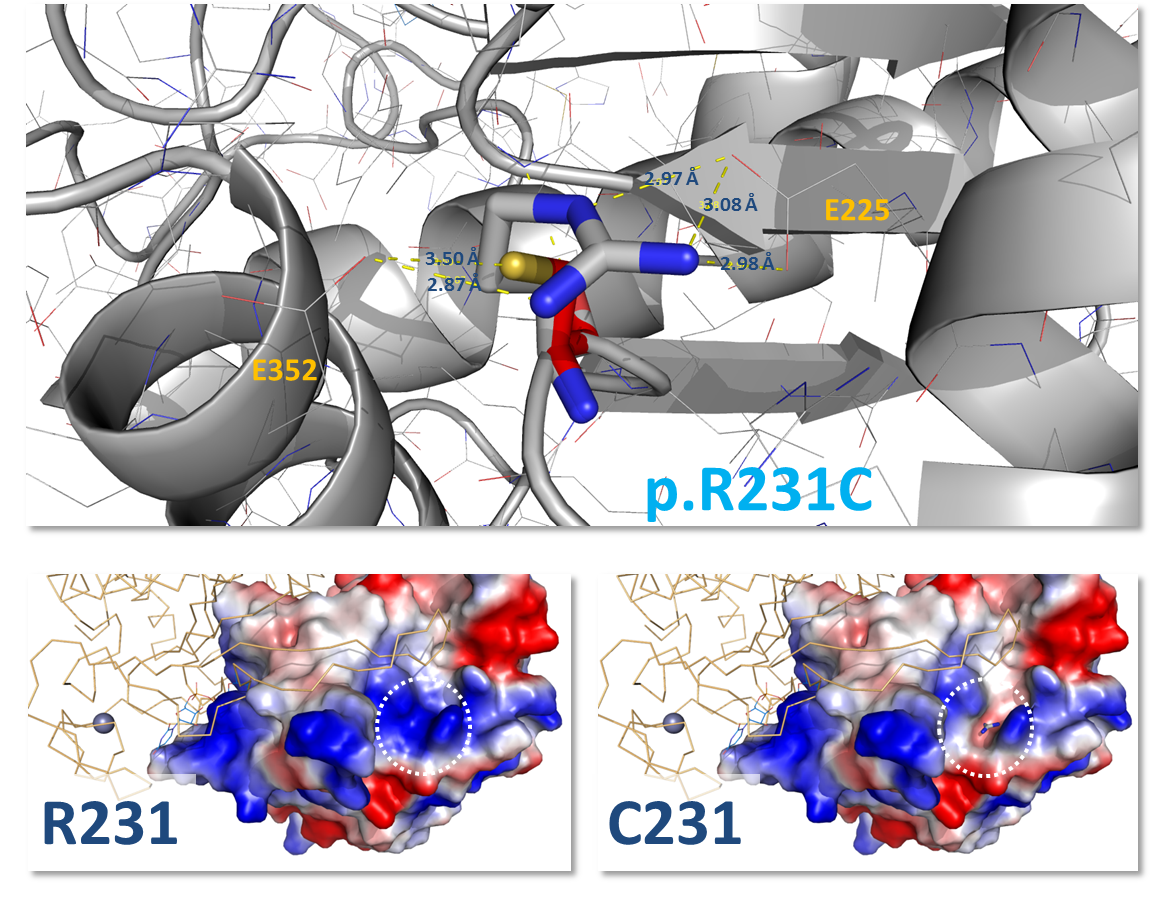 |
| --- |
| **Figure S11 -** Structural impact of the p.R231C mutation in human GALT. Structural model of human p.R231C GALT ( grey cartoon) and opposing monomer from *Escherichia coli* GalT (orange ribbon, PDB code 1GUP). *Top panel*, R231 is represented in grey and the substituting C231 is in red. *Bottom panels*, surface electrostatics map surrounding the R231 residue (*left*) and the substituting C231 residue (*right*); white dotted circles highlight the R231/C231 location. Red, negative charge; blue, positive charge; white, neutral. Figure generated with PyMOL. |

**Figure S11 Coelho (2014)**

| 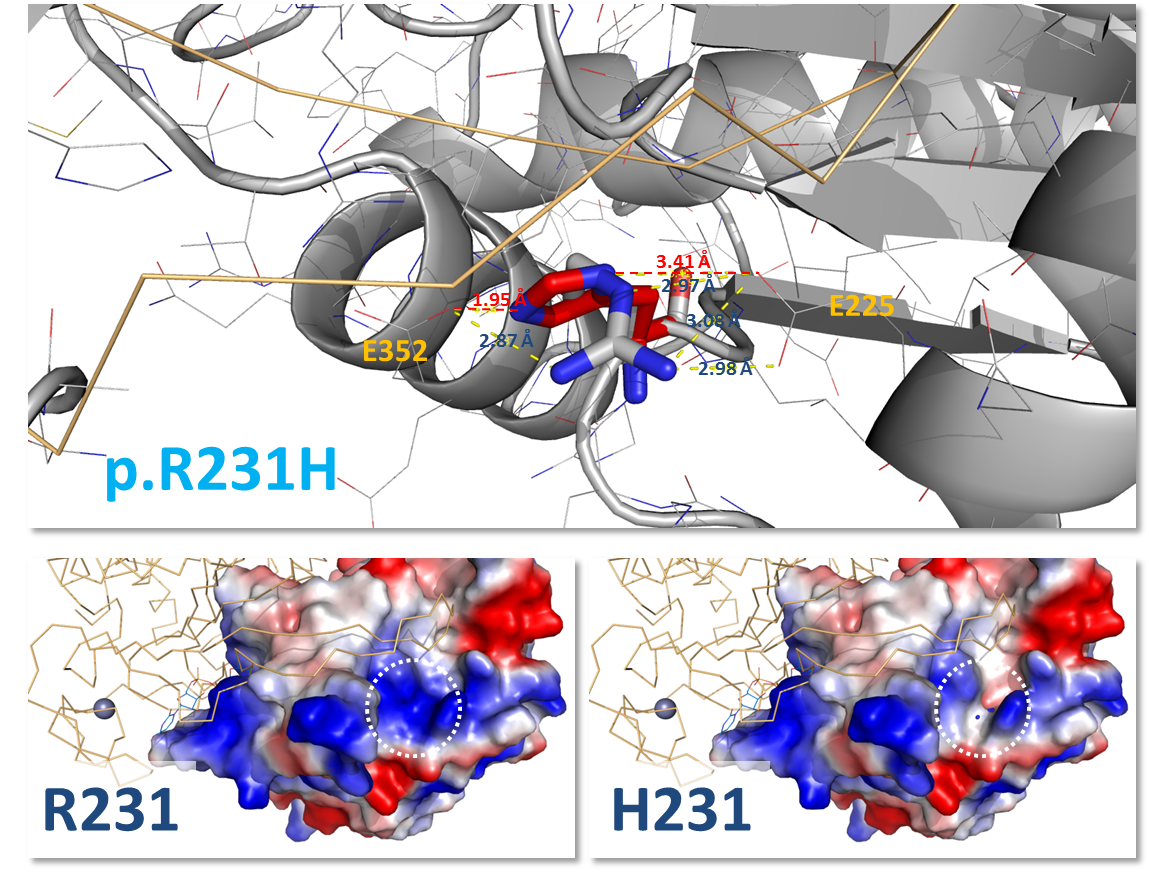 |
| --- |
| **Figure S12 -** Structural impact of the p.R231H mutation in human GALT. Structural model of human p.R231H GALT (grey cartoon) and opposing monomer from *Escherichia coli* GalT (orange ribbon, PDB code 1GUP). *Top panel*, R231 is represented in grey and the substituting H231 is in red. *Bottom panels*, surface electrostatics map surrounding the R231 residue (*left*) and the substituting H231 residue (*right*); white dotted circles highlight the R231/H231 location. Red, negative charge; blue, positive charge; white, neutral. Figure generated with PyMOL. |

**Figure S12 Coelho (2014)**
