# Supplementary material for: Functional and structural impact of the most prevalent missense mutations in classic galactosemia
Source: Mol Genet Genomic Med. 2014 Jun 23;2(6):484–96. doi: 10.1002/mgg3.94 (PMC4303218; doi:10.1002/mgg3.94)
Supplement: Table S1 — Oligonucleotides used for site-directed mutagenesis. [file mgg30002-0484-sd3.docx]

**Table S1**. Oligonucleotides used for site-directed mutagenesis.

| **Primers^a^** | **Sequence (5’→3’)** ^b^ |
| --- | --- |
| Q188R-F | CCC CAC CCC CAC TGC CGG GTA TGG GCC AGC AG |
| Q188R-R | CTG CTG GCC CAT ACC CGG CAG TGG GGG TGG GG |
| S135L -F | GCT TCC ACC CCT GGT TGG ATG TAA CGC TGC |
| S135L-R | GCA GCG TTA CAT CCA ACC AGG GGT GGA AGC |
| K285N-F | GAA GAA GCT CTT GAC CAA TTA TGA CAA CCT CTT TGA G |
| K285N-R | CTC AAA GAG GTT GTC ATA ATT GGT CAA GAG CTT CTT C |
| N314D-F | GGC TGG GGC CAA CTG GGA CCA TTG GCA GCT GC |
| N314D-R | GCA GCT GCC AAT GGT CCC AGT TGG CCC CAG CC |
| R148Q-F | GGT CCC TGA GAT CCA GGC TGT TGT TGA TGC |
| R148Q-R | GCA TCA ACA ACA GCC TGG ATC TCA GGG ACC |
| G175D-F | GCA GAT CTT TGA AAA CAA AGA TGC CAT GAT GGG CTG TTC |
| G175D-R | GAA CAG CCC ATC ATG GCA TCT TTG TTT TCA AAG ATC TGC |
| P185S-F | GCT GTT CTA ACC CCC ACT CCC ACT GCC AGG |
| P185S-R | CCT GGC AGT GGG AGT GGG GGT TAG AAC AGC |
| R231C-F | GCT ACT CAG GAA GGA ATG TCT GGT CCT AAC CAG TG |
| R231C-R | CAC TGG TTA GGA CCA GAC ATT CCT TCC TGA GTA GC |
| R231H-F | GCT ACT CAG GAA GGA ACA TCT GGT CCT AAC CAG TG |
| R231H-R | CAC TGG TTA GGA CCA GAT GTT CCT TCC TGA GTA GC |

^a^ F, forward; R, reverse. ^b^ mutagenesis sites are underlined**.**
