# Supplementary material for: Functional and structural impact of the most prevalent missense mutations in classic galactosemia
Source: Mol Genet Genomic Med. 2014 Jun 23;2(6):484–96. doi: 10.1002/mgg3.94 (PMC4303218; doi:10.1002/mgg3.94)
Supplement: Table S2 — Effect of GALT substrates and putative metal cofactors on the conformational stability of GALT variants. Thermal shifts observed on WT and variant GALT proteins induced by addition of the substrates galactose-1-phosphate (Gal-1-P) and UDP-glucose (UDP-Glc) and the metal ions (Fe2+ and Zn2+), monitored by differential scanning fluorimetry. [file mgg30002-0484-sd4.docx]

**Table S2. Effect of GALT substrates and putative metal cofactors on the conformational stability of GALT variants.** Thermal shifts observed on WT and variant GALT proteins induced by addition of the substrates galactose-1-phosphate (Gal-1-P) and UDP-glucose (UDP-Glc) and the metal ions (Fe^2+^ and Zn^2+^), monitored by differential scanning fluorimetry.

|  | **UDP-Glc ^a^** | | **Gal-1-P ^b^** | | **Fe^2+ c^** | | **Zn^2+ d^** | |
| --- | --- | --- | --- | --- | --- | --- | --- | --- |
|  | **Δ*T*_m1_ (°C)** | **Δ*T*_m2_ (°C)** | **Δ*T*_m1_ (°C)** | **Δ*T*_m2_ (°C)** | **Δ*T*_m1_ (°C)** | **Δ*T*_m2_ (°C)** | **Δ*T*_m1_ (°C)** | **Δ*T*_m2_ (°C)** |
| **WT** | +0.2 | +0.3 | -0.2 | -0.3 | +1.4 | -0.8 | -3.7 (1.7)^e^ | -4.8 (2.1)^e^ |
| **Q188R** | -0.2 | -0.4 | -0.4 | +0.3 | -0.6 | -0.4 | +0.8 | -0.2 |
| **S135L** | +0.4 | -0.1 | +0.1 | +0.1 | +0.6 | -0.1 | -2.5 (1.3)^e^ | -3.4 (0.9)^e^ |
| **K285N** | +0.4 | +0.1 | +0.1 | -0.1 | -1.5 | -0.5 | -3.9 (2.1)^e^ | -5.2 (1.5)^e^ |
| **N314D** | -0.7 | -0.9 | -0.5 | -0.6 | -0.9 | -0.9 | -1.5 | -3.4 (0.9)^e^ |
| **R148Q** | -0.6 | -0.4 | -0.6 | -0.3 | +1.5 | -0.3 | *-2.0 (1.3)*^e,f^ | -2.9 (0.6)^e^ |
| **G175D** | +0.5 | -0.1 | +0.5 | +0.4 | 0.6 | 0.1 | -2.5 (0.3)^e^ | -3.5 (0.1)^e^ |
| **P185S** | -0.9 | +0.1 | +0.1 | +0.1 | +2.5 (0.5)^e^ | -0.5 | -3.4 (1.1)^e^ | -3.7 (0.4)^e^ |
| **R231C** | -0.2 | +0.2 | -0.5 | +0.1 | -1.0 | -0.6 | -5.5 (0.3)^e^ | -5.0 (0.6)^e^ |
| **R231H** | -0.1 | -0.2 | -0.2 | -0.2 | -1.0 | 0 | -5.0 (0.4)^e^ | -4.6 (0.5)^e^ |

^a^ [UDP-Glc] = 0.5 mM. ^b^ [Gal-1-P] = 2 mM. ^c^ [Fe^2+^] = 100 μM. ^d^ [Zn^2+^] = 100 μM. ^e^ standard deviation (SD) values between brackets, for |Δ*T*_m_| values ≥ 2 °C (*n*=3). ^f^ Δ*T*_m_ deemed not significant, since Δ*T*_m_-SD lies below the 2.0 °C threshold.
